# The General Practitioner and Original Research

**Published:** 1907-07-27

**Authors:** 


### The Hospital

A Journal of

The Medical Sciences and Hospital Administration.

New Series. No. 18, Vol. I. [No. 1090, Vol. XLII.] SATURDAY, JULY 27, 1907.

### The General Practitioner and Original Research.

In his recent address delivered at the opening of the Leeds Post-graduate Course, Dr. James Mackenzie, of Burnley, took as his subject the rôle of the general practitioner in the advancement of medical science. The boldness that prompted the choice of so interesting a theme is as praiseworthy as the admirable manner in which the author acquitted himself of his self-imposed task, and general practitioners may well feel grateful that their cause was in such capable hands. To an assembly of general practitioners, indeed, we know of no more instructive, no more encouraging subject for a paper than that of the debt which science owes to the persevering, observant, painstaking doctor who collects the mortar from which specialists build their edifices. Dr. Mackenzie touched but the fringe of the subject. He was silent upon the historical side of the question, not, we take it, because he was unaware of what the general practitioner has actually done towards advancing the science of medicine, but because he preferred, quite justly, to consider the achievements that may yet result from correct observation and faithful, unbiassed recording.

Yet the historical side of the subject is as fascinating as the prospective. The striking example of Jenner (to quote only one) is sufficiently convincing to show that a man needs neither the leisure of the embryonic specialist nor the emoluments of a university fellowship to prosecute, diligently and faithfully, original research with a special, single-minded endeavour to arrive at a certain and tangible result. In the past, and at present, the general practitioner has done, and is doing, his share of work on the temple of truth. The bricks he brought may have been small, flaked bits of plaster in comparison with the marble pilasters which the specialist raised, but they had their value, and have served to strengthen and support the edifice. The true worker finds his tools everywhere, and truth is not necessarily nearer in a well-equipped laboratory than in the surgery of a country practitioner. There is an impression that proper research cannot be carried out in the absence of those aids on which the student and the young specialist rely—the library, the laboratory, the expert to advise and correct, the discussion in scientific journal or learned society. "It seems a hopeless task to the general practitioner to do anything of an important nature in the investigation of disease, and his enthusiasm for his profession is thus damped and his faculties practically paralysed," says Dr. Mackenzie, and most of us will readily agree with him. It is indeed hard for the student, bereft of the props of support to which he looked while at his medical school, to realise, once he is in the unassisted wilds of private practice, that he is able to do something to advance the science which he loves and for which he still feels a certain amount of enthusiasm. Yet there are many who have survived the disappointments that the first knowledge of their apparent impotence brings them, and who have done good work—work, that is, that will stand the test of time as well as that achieved by any expert worker in an up-to-date laboratory. In many cases it is amazing, to the man at least who holds that original research is a matter of the schools, of State-aided enthusiasm, or of grants from associations and societies, to find what has been done by those who had apparently nothing except their native intelligence, their eagerness in the pursuit of truth, and their desire to help forward the profession which they have chosen. Who, for instance, has not been struck with the remarkable number of excellent papers on scientific subjects, often of an important nature, which emanate from general practitioners in Italy? There the general practitioner is eminently an original observer, collecting data, drawing deductions, and formulating in a quiet, unassuming way, theories and speculations which with us are reckoned to be the special privilege of the specialist attached to some well-known medical school.

What the general practitioner needs in pursuing his original researches is a medium for expressing himself, a medium in which he can give to the world, to his colleagues, what he has collected and what he has surmised or proved. The local society affords such a medium, but its range is limited, and the circle to which the observer appeals when he reads his paper there is necessarily a small one. To a large extent the recognised journals of the profession are closed against him. They depend for their contributions on more things than merit. We do not allege that papers of outstanding interest do not stand a chance of publication and discussion. What we do allege is that the average general practitioner feels unequal to the task of braving the preliminaries which are necessary before he can get his claims recognised and, when recognised, appreciated. To help him in his endeavours it is necessary that societies and journals should encourage him to a far greater extent than they do at present. We do, perhaps, overmuch for the student or the recently qualified in comparison with what we hold out to the practical working man whose research is less a bid for fame or for the winning of a scholarship than for an attempt to arrive at honest interpretation of recorded clinical facts. Every inducement should be held out to the latter, not to undertake original research, but to publish or communicate his results when he has, single-handed, embarked upon such a venture. For the future holds much in store for the pure observer as distinct from the theorising expert, and we lay claim to no great gifts of prophecy when we venture to assert that in the advancement of science the general practitioner will of necessity play an important rôle in days to come.

